# Complement Dysregulation in Kidney Diseases: Mechanisms, Biomarkers, and Emerging Targeted Therapies

**DOI:** 10.3390/ijms27083466

**Published:** 2026-04-13

**Authors:** Patryk Jesiołowski, Mateusz Krzywda, Agnieszka Furmańczyk-Zawiska, Magdalena Durlik

**Affiliations:** 1 Department of Transplantology, Immunology, Nephrology and Internal Medicine, Medical University of Warsaw, 02-091 Warsaw, Poland; 2 Transplantation and Nephrology Student Scientific Association, Department of Transplantology, Immunology, Nephrology and Internal Medicine, Medical University of Warsaw, 02-091 Warsaw, Poland

**Keywords:** complement system, kidney diseases, alternative pathway, complement dysregulation, biomarkers, acute kidney injury, C3 glomerulopathy, atypical hemolytic uremic syndrome, IgA nephropathy, complement-targeted therapies

## Abstract

The complement system is the primary defense mechanism against pathogens, acting through opsonization, the membrane attack complex, and classical, lectin, or alternative pathways. These pathways result in the production of key complement components, including C3a (complement component), C5a, and C3b, which recruit inflammatory cells. Complement dysregulation leads to renal disease through the overproduction of anaphylatoxins or inappropriate formation of the membrane attack complex. The levels of complement components have been shown to be useful as predictive markers in acute kidney injury, especially in conditions of alternative pathway activation, and in diseases of immune complex pathology such as lupus nephritis and IgA nephropathy. Genetic defects in complement regulatory proteins result in diseases such as C3 glomerulopathy or atypical hemolytic uremic syndrome, in which uncontrolled C3 convertase activity results in renal failure. Therapeutic interventions targeting complement components, including eculizumab or pegcetacoplan, improve patient outcomes in atypical hemolytic uremic syndrome and C3 glomerulopathy, respectively, while other interventions improve renal function in IgA nephropathy. These findings underscore the dual role of the complement system, which is not only implicated in the progression of renal diseases but also provides the potential for the development of therapeutic interventions for the treatment of various forms of nephropathy.

## 1. Introduction

The complement system is a key component of innate immunity and a clinically relevant mediator of kidney injury when dysregulated [1,2,3]. In renal diseases such as acute kidney injury (AKI), lupus nephritis (LN), C3 glomerulopathy (C3G), atypical hemolytic uremic syndrome (aHUS), IgA nephropathy (IgAN), and diabetic kidney disease (DKD), aberrant complement activation, particularly of the alternative pathway, plays a key role in disease pathogenesis [2,3,4,5]. This dysregulation contributes to tissue injury through the generation of proinflammatory mediators (C3a and C5a), opsonins (C3b), and the membrane attack complex (MAC, C5b-9). Furthermore, studies have indicated that urinary complement components may be more representative of intrarenal inflammation than traditional serum markers [6]. Accordingly, this review follows a translational approach to link complement dysregulation to kidney diseases. First, we discuss the pathogenic mechanisms by which complement dysregulation contributes to kidney diseases. Furthermore, we highlight biomarkers that may improve diagnosis, prognostication, and PD (pharmacodynamic) assessment. Finally, we summarize emerging complement-targeted therapies and their implications for personalized medicine approaches for complement-mediated kidney diseases.

## 2. Mechanisms of Complement Activation

The complement system is activated through three pathways: classical (CP), lectin (LP), and alternative (AP). C3 serves as the central amplification point in this cascade, and its activation requires strict regulation. Excessive C3 cleavage results in uncontrolled opsonization and inflammation [7,8,9]. C1q initiates the classical pathway by binding to IgG or IgM antibodies attached to antigens [10,11]. This interaction activates C1r and subsequently C1s in a calcium-dependent manner [11,12]. C1s cleaves C2, and the C2a fragment binds to C4b to form the classical C3 convertase, C4b2a. The released Ba (factor B fragment may limit excessive activation and restrict convertase assembly to the site of activation [13]. Ba represents a potential marker of alternative pathway activity, although its use in routine practice remains limited. Measurement requires strict pre-analytical handling, including rapid cooling and prompt processing, and relies on specialized methods such as ELISA or mass spectrometry [6,14,15]. At present, Ba should be regarded as a research biomarker requiring further validation in urinary complement studies. In the lectin pathway, C1s-mediated cleavage results in covalent binding of C4b to the cell surface. This process localizes complement activation to the target site [16,17]. This pathway shares the same downstream convertase as the classical pathway. It is activated by MBL (mannose-binding lectin), collectins, and ficolins in association with MASPs (mannose-binding lectin-associated serine proteases), which recognize carbohydrate patterns on microbial surfaces [18,19,20]. The alternative pathway begins with the spontaneous hydrolysis of C3 to C3(H_2_O). Factor D cleaves factor B, generating the fluid-phase C3 convertase [21]. On cell surfaces, C3b binds factor B and forms C3bBb, which is stabilized by properdin and enhances its activity on the target surface [21,22]. Measurement of Bb (catalytic subunit of factor B) requires standardization, as studies have used different assay platforms in plasma and serum. Bb should therefore be considered a translational biomarker rather than a routine clinical test [6,23].

Factor H (CFH/FH), a key regulator encoded by the *CFH* gene, and factor I (CFI/FI), a regulator encoded by the *CFI* gene, are the main regulators of this pathway. Factor H binds host surface polyanions and accelerates the decay of alternative pathway convertases. Factor I cleaves C3b and C4b in the presence of cofactors such as factor H, C4BP, CR1, or CD46 [24,25]. This regulatory mechanism is critical in tissues exposed to continuous complement activity, including the kidney and microvascular endothelium. Impaired regulation promotes chronic inflammation and tissue injury [26]. C3d and C3c are degradation products of C3b and reflect complement activation regardless of the initiating pathway. Their measurement lacks standardization, and testing remains limited to specialized laboratories [15,27]. These fragments should be regarded as research biomarkers pending further validation in kidney disease.

High levels of C3b promote formation of C5 convertases, C4b2a3b and C3bBbC3b from C3 convertases [28]. These complexes cleave C5 into C5a and C5b. C5b initiates formation of the membrane attack complex through sequential binding of C6, C7, C8, and C9, resulting in pore formation and cell lysis [29]. sC5b-9 (soluble terminal complex) reflects activation of the terminal complement pathway, although its clinical application remains limited. Variability in sample collection, handling, and laboratory methods affects reproducibility [15,30]. It should therefore be considered a promising translational biomarker rather than a fully validated standalone marker of renal complement deposition. However, plasma levels do not consistently correlate with kidney TCC (Transitional Cell Carcinoma) staining [31].

## 3. Regulation and Homeostasis of the Complement System

The activation of the complement system is regulated by various soluble and cell surface regulators that maintain the host homeostasis. They suppress the assembly of convertase enzymes, promote their decay, and mediate proteolytic inactivation of C3b/C4b on host cell surfaces [32]. In contrast, the aforementioned fluid-phase complement regulator factor H is the major inhibitor of the alternative pathway. It accelerates the decay of the C3 component of the alternative pathway (AP) C3 convertase complex. FH also functions as an essential cofactor for the proteolytic inactivation of C3 by the enzyme factor I [33]. The detection of anti-FH antibodies and C3 nephritic factor (C3NeF) remains challenging, particularly due to the lack of standardization of the C3NeF assay [34]. Their availability in routine practice is limited due to the need for specialized laboratories, as well as the relatively high cost associated with complex functional assays [35]. Although clinically relevant in the context of C3 glomerulopathy and aHUS, these autoantibodies should be considered as functionally incompletely validated. Membrane-bound regulators function as a secondary line of defense. The decay-accelerating factor (DAF), also known as CD55, destabilizes preformed C3/C5 convertases by accelerating the dissociation of enzymatic subunits [36]. The membrane cofactor protein (MCP), also known as CD46, functions as a cofactor for factor I and enables cleavage of C3b/C4b in the cell-bound form to protect against the opsonization of the host [37,38]. CD59 glycoprotein is a glycophosphatidylinositol-anchored inhibitor of the assembly of C5b-8/C5b-9 complexes. CD59 binds these complexes on the cell surface and prevents C9 insertion and polymerization, thereby protecting target cells, including renal vascular and parenchymal cells, from membrane attack complex–mediated lysis [39]. C1-inhibitor (C1-INH) regulates the initiation of both classical and lectin pathways. It binds covalently and noncovalently to activated C1r/C1s and MASP proteases (MASP-1 and MASP-2). Analysis of C1-INH-serine protease complexes has indicated its function in regulating the classical pathway and lectin pathway (CP/LP) in human plasma [40,41]. Functionally, the amplification loop of the alternative pathway is the prominent driver of C3 and, consequently, C5 cleavage in the fluid phase. Experimental studies have demonstrated that the amplified pathway contributes to the flux through the terminal pathway. Thus, the amplification pathway was proposed to provide C3/C5 cleavage via the classical and lectin pathways [28]. At the kidney level, recurrent mutations occur in genes encoding regulatory proteins (e.g., CFH glycoprotein or CD46/MCP) or complement enhancers (e.g., gain-of-function mutations in C3 or factor B). These mutations are observed in patients with aHUS and may also influence the phenotype of C3 glomerulopathy (C3G) [42]. At the molecular level, CFH mutations and structural variations in the *CFH* and *CFHR* region may impair the recognition of polyanionic host ligands or disrupt FH interaction with C3b. This leads to loss of local regulatory function and promotes uncontrolled convertase activity on renal endothelium [43,44,45]. Among acquired defects, the production of high-titer anti-factor H antibodies most closely phenocopies FH deficiency. There are studies that mark the beginning of the demonstration of the importance of anti-factor H in the causation of aHUS [46]. This involves the interaction between a genetic predisposition (e.g., a heterozygous variant in a regulatory gene) and an acquired trigger, such as autoantibody production or an infectious-inflammatory insult. Together, these factors override normal complement regulation [46,47].

Biomarkers used in the diagnosis of complement-associated kidney diseases are summarized in Table 1.

## 4. Pathogenesis of Complement-Dependent Kidney Diseases

### 4.1. Acute Kidney Injury (AKI)

Acute kidney injury (AKI) is the subject of numerous clinical studies because of its association with significant short- and long-term morbidity and mortality, as recent research has shown in hospitalized patients. Beyond hemodynamic and nephrotoxic factors, evidence suggests that uncontrolled activation of the alternative complement pathway contributes substantially to the pathogenesis of AKI in ischemia/reperfusion and sepsis [48]. However, the relative contribution of complement activation compared to other inflammatory pathways remains difficult to quantify in heterogeneous clinical populations. Among cytokines, interleukin-6 (IL-6) is a critical mediator of AKI. Renal ischemia and toxicity induce IL-6 expression in tubular epithelial cells and leukocytes. Furthermore, IL-6 activates IL-6R/sIL-6R trans-signaling and STAT3, which enhances neutrophil infiltration and inflammatory damage. In an experimental model of AKI, serum levels of sIL-6R were found to be increased approximately threefold, whereas IL-6-deficient mice were resistant to AKI [49,50]. This highlights that cytokine-driven pathways may operate in parallel with complement activation rather than solely downstream. The role of IL-17A in mediating AKI in sepsis was also evident because it was found to induce a pro-inflammatory cytokine and chemokine gene expression profile. Moreover, IL-17A induces apoptosis in tubular epithelial cells through intrinsic caspase-3 activation and the Bax-Bcl-2 pathway. In a cohort of 146 patients with sepsis-induced AKI (62 patients) and without AKI (84 patients), IL-17A was significantly elevated in patients with AKI compared to those without AKI. Furthermore, it also predicted AKI stages and mortality (AUC 0.811; cut-off 4.7 pg/mL; sensitivity 77.4%, specificity 71%) [51,52]. Its predictive value, however, may be affected by systemic inflammation, complicating interpretation in critically ill patients. IL-18 is produced during renal ischemia/reperfusion and mediates tubular injury and inflammation. In IL-18–deficient mice, AKI severity was reduced, with improved renal function and lower neutrophil/macrophage infiltration. In pediatric cardiac surgery patients, urinary IL-18 >75 pg/mg predicted AKI with 88% sensitivity [53]. This shows that IL-18 may be an early signal of tubular injury, but its performance is not uniform across populations. This biomarker seems more reliable in children than in adults. However, urinary IL-18 has not been uniformly predictive in adults. One study of 100 cardiac surgery patients showed an AUC of 0.53–0.55, suggesting urinary IL-18 may reflect systemic inflammation rather than kidney-specific injury [54]. In mouse studies with ischemic-reperfusion injury, the deposition of C3 in the kidney tissue occurs before the onset of the histological changes in tubular cell necrosis. Inhibition of the proximal pathway of the complement system or disruption of the *C3* gene greatly reduces the severity of the injury [55]. Nevertheless, translating these findings into human disease remains challenging due to differences between controlled experimental models and complex clinical conditions. Disruption of C5a-C5aR1 signaling in preclinical reperfusion and septic models resulted in decreased apoptosis in tubular cells, maintained cortical microcirculatory flow rates, and decreased inflammatory cell infiltration [56]. This suggests a potentially important therapeutic target, although clinical validation in AKI populations is still lacking. In human studies of perioperative acute kidney injury (AKI) following cardiac surgery, urinary levels of Ba progressively increase with increasing severity of AKI, preceding elevations in plasma creatinine by several hours [14]. Among the prospective studies of patients in the perioperative period, the patients with acute kidney injury (AKI) had higher concentrations of urinary factor B fragment (Ba). A twofold increase in the concentrations of urinary Ba was found to result in an increased risk of AKI, implying that urinary Ba is a useful biomarker for AKI [57]. However, outside the perioperative period, urinary Ba has only been partially validated as a biomarker. Elevated Ba may reflect systemic alternative pathway activation and urine protein passage. Most studies are small and highly selected, limiting statistical power and generalizability. Thus, the clinical value of Ba depends on context, being more informative perioperatively than in broader ICU (Intensive Care Unit) populations [58,59]. It appears to be more informative in perioperative patients than in broader ICU populations. In severe systemic inflammation, its specificity for kidney injury is reduced. In a study of critically ill children, the original validation cohort was composed of only 73 mechanically ventilated patients, and 56 (76.6%) of them already had AKI. Urine Ba was associated with AKI in this population (adjusted OR 1.57), and only 14 of the 56 AKI patients (25.0%) had stage 3 AKI. This suggests that the highest levels of urinary Ba result from a narrow subgroup. Notably, urinary Ba levels were highest in patients with sepsis-associated AKI, suggesting that systemic inflammatory activation of the complement pathway may amplify the biomarker signal. As a result, elevated Ba levels may reflect systemic complement activation rather than kidney-specific injury [57]. In the case of cytokines, urinary IL-18 rises before serum creatinine and is currently one of the best-studied early cytokine biomarkers of AKI [60]. In another pediatric severe-AKI study, just 14 stage 3 AKI patients were matched to 14 controls, and 5/14 (35.7%) required renal replacement therapy, underscoring the limited precision of the available data [61]. In adults, the ICU cohort consisted of 439 patients. Among them, 252 (57.4%) had no AKI, 124 (28.2%) had stage 1 AKI, 43 (9.8%) had stage 2 AKI, and 20 (4.6%) had stage 3 AKI. Urine Ba was associated with persistent AKI (adjusted OR 6.6), but the study was restricted to the ICU population. In addition, persistent AKI can only be defined in patients who have been in the ICU for more than 48 h [59]. This further limits the applicability of these findings to less severe or non-critical care settings. Multivariate analyses show that normal C3/high sC5b-9 or low C3/normal sC5b-9 independently predict worse kidney outcomes (relative risk 3.7–8) [62]. Furthermore, studies identify two hypoxia-related mechanisms of alternative pathway activation in AKI. First, tubular epithelium upregulates C3 and factor B in response to injury, and second, regulatory proteins (such as CD55/DAF and CD46/MCP) are shed from the injured epithelium [63]. Downstream C5a made in the local region activates C5aR1 on the endothelium. It further activates innate immune cells to initiate signaling cascades through either Gi/Gβγ (phosphoinositide 3-kinase (PI3K) to Akt signaling) or the phospholipase C beta complex (PLCβ/IP_3_/Ca^2+^). Taken together, these mechanisms suggest that complement activation in AKI is closely integrated with broader inflammatory networks rather than acting as an isolated pathway. Additionally, C5a enhances the transcription of chemokine messenger RNA through the NF-kB pathway [64]. Overall, evidence from AKI studies supports complement activation as an early indicator of injury. However, the clinical utility of individual biomarkers is limited by context-specific performance, lack of external validation, and the potential for some signals to reflect systemic inflammation rather than kidney-specific damage.

### 4.2. Lupus Nephritis (LN)

Lupus is a serious and common complication of SLE (Systemic Lupus Erythematosus), contributing significantly to morbidity [65]. Immune complex deposition in the glomeruli classically activates the C1q-C4-C2 axis. However, this classical pathway activation rarely occurs in isolation, as amplification through the alternative pathway substantially modifies the overall inflammatory response. Biopsy staining for C4d and C1q in the tissue corresponds to the level of histopathologic activity and immune complex burden [66]. Intrarenal alternative pathway amplification contributes substantially to C3 cleavage product in active LN, despite the presence of the activated classical pathway. This interaction between pathways complicates the interpretation of complement biomarkers, as they may reflect overlapping mechanisms rather than distinct processes. Persistent complement activation in lupus nephritis drives glomerular injury. Clinically, low circulating C3 and C4, reflecting ongoing consumption, correlate with disease activity and are incorporated into risk scores [67]. Nevertheless, their role as indirect markers of complement activity limits their sensitivity for detecting ongoing intrarenal inflammation. Urinary IL-6 levels are elevated in patients with active lupus nephritis, reflecting ongoing intrarenal inflammation. Increased IL-6 concentrations were detected in 83% of patients (24/29), with significantly higher levels observed in class IV nephritis (*p* < 0.01). Following treatment, urinary IL-6 levels decreased significantly (*p* < 0.001), indicating a response to therapy [68]. This suggests that cytokine markers may be useful for monitoring treatment response, but they lack specificity for complement-mediated mechanisms. The role of IL-6 is also indicated by its ability to induce B cell differentiation and T cell polarization, thereby promoting immune complex formation and complement activation [69]. IL-17A, a pro-inflammatory cytokine, is also a marker of lupus nephritis progression through a Th17 response. It was indicated by increased Th17 levels (1.2% vs. 0.6%, *p* < 0.01), decreased Treg/Th17 ratio, and strong correlations of IL-17A with disease activity (r = 0.561, *p* < 0.001) and complement consumption (C3: r = −0.455, *p* = 0.003). As a marker, IL-17A achieved 75% sensitivity and 76.7% specificity for LN at a defined cutoff [70]. Thus, cytokine-based markers may reflect immune activation more broadly rather than complement-specific disease activity. Additionally, more significant correlations have been shown by alternatively amplified products, such as Bb in serum or C3 fragments in tissue, compared to serum C3/C4 levels in lupus nephritis (LN) patients [71]. This supports the concept that downstream or pathway-specific markers may better capture active disease processes than traditional systemic measurements. Improved clinical outcomes have also been shown in studies focusing on cell-bound complement activation products (CB-CAPs). Cell-bound complement activation products (CB-CAPs), including erythrocyte-bound C4d (EC4d) and B cell-bound C4d (BC4d), outperform traditional C3/C4 measurements, correlating more strongly with disease activity [72]. CB-CAPs appear to capture downstream complement activation more directly than routine serum C3 and C4 measurements. This suggests that routine complement tests may miss ongoing disease activity. Downstream products may better reflect the current inflammatory state of the kidney. For this reason, CB-CAPs may have greater clinical value in monitoring LN. In clinical practice, serum C3 and C4 levels are currently used for the monitoring of SLE patients, but the usefulness of this approach is limited, as C3 and C4 act as a substrate, not as a product, of the complement pathway. Furthermore, they may not fully capture ongoing complement activation [73]. In the context of LN, C4d in the plasma of SLE patients with LN was found to be significantly higher than in SLE patients without LN (1.02 vs. 0.57 mg/L, respectively; *p* = 0.004). In addition, the C4d/C4 ratio in SLE with LN was also found to be higher than in SLE without LN (11.2 vs. 2.5, respectively; *p* = 0.0002). The C4d/C4 ratio had higher specificity, sensitivity, and accuracy for LN than C4d and C4 [74]. This indicates that combining activation products with substrate measurements may improve diagnostic performance compared to either alone. In 71 LN patients followed for a mean of 35 months, renal flares were identified with only 75% sensitivity/71% specificity for C3 and 48% sensitivity/71% specificity for C4, and neither marker fell before the flare [75]. This limits the usefulness of C3 and C4 as early warning markers. Their decline may reflect established activity rather than an impending flare. More direct markers of complement activation may therefore be more useful. By contrast, in a pilot LN study, patients with nephritis had higher erythrocyte-bound C4d and reticulocyte-bound C4d than both non-SLE renal controls and SLE controls without nephritis, and erythrocyte-bound C4d correlated with the NIH activity index (r = 0.55, *p* = 0.04) [76]. Furthermore, plasma C4d has been shown to outperform conventional complement markers, identifying nephritis with a sensitivity of 79%, while high C4d levels combined with anti-dsDNA antibodies predicted future LN (OR 5.4, 95% CI 1.4–21.3). Importantly, high C4d demonstrated significantly better diagnostic performance than low C4 (*p* = 0.002) and a comparable performance to low C3 [77]. A cohort study by Toy et al. showed that an elastase-sensitive *C5* polymorphism (rs17611, 2404G>A) plays a role in lupus nephritis, as this polymorphism correlated significantly with increases in urinary C5a and membrane attack complex levels during LN flare-ups, particularly in 2404-GG genotype individuals [78]. Moreover, an increase in urinary and plasma split product levels (C5a, sC5b-9, and Bb) correlates with proliferative lesions and has been related to the reduced kidney response in studies of conventional immunosuppression [71]. This further supports the idea that complement activation is linked not only to disease activity but also to treatment resistance. In lupus nephritis, the strongest evidence appears to favor markers that capture active complement consumption or cell-bound activation, whereas conventional serum C3 and C4 remain imperfect surrogates that may miss ongoing intrarenal amplification.

### 4.3. C3 Glomerulopathy

C3 glomerulopathy (C3G), including dense deposit disease (DDD) and C3 glomerulonephritis (C3GN), is defined by dominant glomerular C3 deposition with minimal immunoglobulin presence. Clinical presentation ranges from microscopic hematuria to nephrotic or rapidly progressive nephritic syndrome [79]. The pathogenesis of C3G involves chronic dysregulation of the alternative complement pathway, with activation occurring within the glomerulus [80]. Although direct interleukin data in C3G are limited, the overactivated alternative pathway produces C3a/C5a-mediated inflammation in the glomerular microenvironment, creating conditions in which cytokines can further enhance leukocyte recruitment and tissue damage [81]. This highlights that current assumptions about cytokine involvement are largely extrapolated rather than disease-specific. Among cytokines, IL-17A is most likely implicated in the pathogenesis of glomerular disease. It stimulates the production of proinflammatory cytokines and chemokines in renal cells and increases neutrophil infiltration. Furthermore, IL-17A is also associated with experimental autoimmune glomerulonephritis [82]. However, its direct contribution to human C3G remains insufficiently validated. IL-6 can support mesangial and tubular cell inflammation and fibrosis through the gp130/JAK/STAT3 pathway [83]. The diagnostic criteria for C3G include positive immunohistochemistry for C3, exceeding all other immune reactants by at least two logarithmic orders of magnitude, with negative or very weak staining for C1q, IgG, IgA, and IgM. In C3GN, electron-dense deposits are typically mesangial and/or subendothelial, whereas in DDD, intensely electron-dense deposits occur within the glomerular basement membrane. These ultrastructural differences reflect activation of the alternative complement pathway [84,85]. C3G patients often have decreased serum C3 levels due to continuous activation of the complement system. In a study by Michels et al. that included 29 biopsy-proven pediatric patients with C3 glomerulopathy, 19 (65%) were classified as having DDD, while 10 (35%) were classified as having C3GN. Low serum C3 level at diagnosis was found in approximately 84% of all patients, including both DDD and C3GN patients. Moreover, all patients showed dominant C3 deposition in kidney biopsies according to the diagnostic criteria for C3G [86]. Large-scale studies show that many C3G patients harbor acquired drivers (autoantibodies like C3 nephritic factor or anti-factor H), while others carry rare pathogenic variants in complement genes (*CFH*, *CFI*, *C3*, *CFB*), emphasizing the co-existence of both [87]. Functionally assayed in C3G patients, the nephritic factor present in the blood or anti-factor H antibodies can stabilize the C3bBb convertase. They may also interfere with the regulatory function of factor H (FH). As a result, the normal control of the complement pathway is disrupted. This leads to reduced serum C3 levels and promotes the accumulation of C3 in the glomeruli [34]. Phenotypic heterogeneity in C3G is better explained by differences in complement endotypes than by morphology alone. This means that similar biopsy findings can come from different mechanisms. A single histologic label does not capture the full disease biology. In a 886-patient cohort, 48% clustered into autoantibody-driven disease and 43% remained without an identifiable genetic and/or acquired driver, while an independent 398-patient series demonstrated rare *CFH*, *CFI*, or *C3* variants in 17% of cases [88,89]. Complement biomarker patterns are often discordant: 25% show isolated convertase dysregulation, 37% have low C3 with high sC5b-9, and 37% show normal C3 and sC5b-9, highlighting that similar biopsy phenotypes can reflect very different levels of alternative pathway activation [89]. Biomarker genotype composite scores (estimated glomerular filtration rate (eGFR), presence of proteinuria, and levels of complement autoantibodies) provide risk stratification for the progression to kidney failure in adult patients [87]. Since the primary injury occurs via alternative pathway activation on the glomerular surface, combining proximal AP inhibitors with direct C3 blockers (factor B/D or C3 inhibitors) is a rational therapeutic approach [90]. Variable response to C5 blockade in C3G reflects heterogeneity of the alternative pathway rather than drug effects. Herlitz et al. found that glomerular C3 deposition persisted in eculizumab-treated C3G patients. Similarly, Gurkan et al. found that C3G patients treated with eculizumab exhibited ongoing C3 convertase activity. Thus, it shows that inhibition at the C3 level is more closely related to C3G disease mechanisms [91,92]. This suggests that targeting upstream complement activation may be more mechanistically appropriate than terminal pathway inhibition. In a registry-based study of French and Canadian cohorts, Le Quintrec et al. found that 6 of 26 (23%) C3G patients exhibited a global response to eculizumab, 6 of 26 (23%) exhibited a partial response, and 14 of 26 (54%) exhibited no response. The responders had lower baseline eGFR, a more rapidly progressive course of disease, and more extracapillary proliferation on renal biopsy [93]. Serum levels of C3, C3Nef, soluble C5b-9, and complement gene haplotypes did not vary significantly between responders and nonresponders. This shows that available biomarkers do not reliably predict treatment response in all patients. This limits the use of these markers for selecting therapy. Therefore, these complement levels are not useful in predicting response to eculizumab [93]. Similarly, in a German study, only 5 of 11 C3G patients exhibited stable renal function on eculizumab, while 6 of 11 exhibited poor outcomes. The authors concluded that the benefits of eculizumab in chronic progressive C3G are limited [94]. Mechanistically, although C5 blockade suppresses terminal complement activation, it does not correct upstream alternative pathway dysregulation. Therefore, terminal pathway inhibition may be incomplete in C3G, and the main disease process can remain active even when C5 is blocked. This helps explain the variable response to eculizumab.

### 4.4. Atypical Hemolytic Uremic Syndrome (aHUS)

Atypical Hemolytic Uremic Syndrome (aHUS) is characterized by complement-mediated thrombotic microangiopathy caused by uncontrolled activation of the alternative complement pathway on the surface of endothelial cells. This results in unregulated convertase activity, C5a generation, and distal glomerular filtration impairment [95]. This indicates that aHUS is driven by both complement activation and endothelial injury. The kidney damage is not only a consequence of thrombosis, but also of ongoing inflammatory activation. In complement-mediated aHUS, pro-inflammatory cytokines including IL-6, IL-1β, and TNF-α have been found to be elevated in patients and murine models, reinforcing the suggestion that pro-inflammatory “second hits” may exacerbate endothelial damage in addition to abnormalities in the alternative complement pathway [96,97]. This suggests that genetic risk is not sufficient on its own. Clinical triggers likely help determine when the disease becomes overt. The inflammatory environment may therefore shape disease severity. In an exploratory cohort of patients with aHUS, soluble TNF-R1 was elevated at baseline in 100% of patients and normalized with eculizumab treatment. Meanwhile, C5a was found to upregulate TNF-α and VCAM-1, suggesting a self-perpetuating effect of complement activation and endothelial activation/cytokine production [98]. In patients with atypical hemolytic uremic syndrome (aHUS), the most common presentation is a combination of microangiopathic hemolytic anemia, thrombocytopenia, and acute kidney injury. Other manifestations include hypertension, proteinuria, kidney dysfunction, and possible compromise of other organ systems such as the neurological, gastrointestinal, and cardiac systems [99]. Genetic variants in complement regulators or alternative pathway components (CFH, CFI, MCP/CD46, C3, CFB) are present in most patients, with CFH being the most frequent and clinically relevant. *CFH* represents the most prevalent and clinically significant genotype variation [100]. In a retrospective study by Mocanu et al., approximately 74% of aHUS patients with aHUS carried variants in complement genes, with *CFH* mutations present in ~39%, *CFI* in ~22%, *C3* in ~13%, and *CD46* in ~9% of individuals [101]. Another study showed that 30% of individuals with aHUS had at least one abnormal variant or anti-CFH. The study also showed that alterations in *CFH*, *C3*, and *CD46* were present in 11%, 5.5%, and 4.6%, respectively. The studies indicate that complement abnormalities play a significant role in the pathogenesis of aHUS [102]. Phenotypic heterogeneity is significant. In the Global aHUS Registry, the male-to-female ratio was 1.3:1 in childhood-onset and 1:2 in adult-onset cases, with 19–38% experiencing extrarenal manifestations within 6 months [103]. Similarly, the response to terminal C5 blockade is heterogeneous. In a study where patients were matched for genotype, 5-year ESKD-free survival increased from 39.5% to 85.5% (hazard ratio 4.95, 95% CI 2.75–8.90, *p* < 0.001, NNT 2.17) for patients treated with eculizumab. The treatment effect was associated with the underlying genotype [104]. This phenotypic variability is further illustrated by studies in which anti-C5 therapy was discontinued. 22% of the 151 patients experienced a recurrence of TMA, and 8% of these progressed to end-stage renal disease (ESRD) [105]. This shows that complement blockade does not remove the underlying risk in every patient. Some patients remain vulnerable to relapse after treatment stops. The clinical course is therefore heterogeneous even within the same diagnosis. Another Brazilian cohort reported relapse risks of 34%, 44.5%, and 58% at 114, 150, and 397 days, respectively [106]. The baseline levels of soluble C5b-9 in plasma, which indicate terminal pathway activation, have been shown to be associated with the level of dialysis requirements [107]. In aHUS, complement dysregulation is highly actionable, but the heterogeneity of genotype, trigger exposure, and relapse risk shows that treatment decisions should integrate more than the diagnosis alone, especially when considering withdrawal or long-term blockade.

### 4.5. IgA Nephropathy (IgAN)

IgA nephropathy is the most common primary glomerulonephritis worldwide. The disease process is initiated by the deposition of galactose-deficient IgA1 (Gd-IgA1) in the mesangium [108]. The immune complex activates the complement system via the lectin and alternative pathways in the mesangium and the glomerular capillary wall, resulting in the production of C3 fragments, C5a, and C5b-9 [109]. Aside from activation of the terminal pathway, the study findings suggest that lectin and alternative pathway activation may both be implicated in IgA nephropathy, but in different ways. Moreover, lectin pathway activation in the glomerulus has been associated with more severe renal disease in a classic biopsy study. Studies have shown that urinary C4d and MBL levels correlate with crescent formation and disease progression in crescentic IgAN [110,111,112]. These findings suggest that lectin pathway markers are linked to more aggressive disease. Their association with crescent formation makes them more useful as activity markers than as simple diagnostic markers. At the same time, alternative pathway activation was associated with clinical severity of IgAN, including a correlation of circulating factor B/Bb levels with IgAN severity, Gd-IgA1, vascular lesions, and patient outcomes [113,114]. This suggests that alternative pathway activity may reflect broader disease burden. It may also connect immune activation with structural kidney injury and worse outcomes. IL-6 functions as an “upstream amplifier” in IgAN, promoting production of galactose-deficient IgA1. In a prognostic cohort of 762 patients, urinary IL-6 levels distinguished IgAN patients from healthy controls with an AUC of 0.9725. This shows that IL-6 may be a strong marker of disease presence. However, diagnostic performance alone does not prove that it is specific for progression. Its main value may be in identifying active inflammatory disease. In a cohort of 762 patients, urinary IL-6 distinguished IgAN patients from healthy controls with an AUC of 0.9725 and independently predicted disease progression (HR 1.420), with 11.3% reaching a composite renal endpoint [115]. The Th17/IL-17 axis also plays a role in active IgAN [116,117]. In a cohort study of patients with IgA nephropathy, an increased proportion of peripheral Th17 cells and elevated serum IL-17A levels were observed compared with healthy controls. Moreover, IL-17A levels positively correlated with the degree of proteinuria and indicators of disease severity [118]. This supports the view that IgAN is not only an immune-complex disease, but it also involves a cytokine-driven inflammatory response. That response may contribute to ongoing renal injury. In IgA nephropathy, the deposition of components of the lectin complement pathway, i.e., C4d, MBL, and MASP-1/2, is found in a substantial proportion of patients and is correlated to the extent of renal damage and prognosis [110,119]. Additionally, C4d staining in the glomerular layer, reflecting a lectin/classical split pattern and associated with C5b-9 deposition, has been linked to severe histopathological injury in recent studies [120]. Increased levels of alternative pathway fragments in the plasma and urine of patients with progressive IgA nephropathy have been found to be elevated and include Bb and C5b-9 [121]. Overall, IgAN appears to involve both upstream immune triggers and downstream complement amplification. This makes the disease biologically heterogeneous. It also explains why a single biomarker is unlikely to capture all clinically relevant phenotypes. The main critical point is that pathway activity does not yet map cleanly onto a single therapeutic sequence, so complement blockade should be framed as phenotype-guided adjunctive therapy rather than universal treatment.

### 4.6. Acute Post-Infectious GN (Glomerulonephritis)

Even though the classical pathway is the recognized stimulus in post-infectious glomerulonephritis (PIGN), recent immunopathological studies indicate the common glomerular accumulation of lectin pathway pattern recognition molecules (MBL and MASPs) and dense terminal complexes (C5b-9) in acute inflammation [122,123]. This suggests that the complement response in PIGN is more complex than classical pathway activation alone. Lectin and terminal pathway products may reflect amplification of the initial immune response. They may also help explain the severity of glomerular injury. Pro-inflammatory cytokine responses, including the Th17/IL-17 axis, may further intensify glomerular inflammation and prolong the proliferative lesion [82]. This indicates that inflammation in PIGN is not only complement-driven. Cytokines may sustain tissue injury after the infection has started to resolve. This may contribute to prolonged histologic activity. In pediatric PSGN (Post- Streptococcal Glomerulonephritis), levels of IL-6 are elevated in the acute phase and may play a role in the systemic amplification of glomerular inflammation. In a study of 12 children, IL-6 levels were significantly higher in patients with PIGN (12.4 ± 4 pg/mL) compared to controls (2.57 ± 0.34 pg/mL, *p* < 0.05), supporting its role as a marker of acute inflammation rather than kidney-specific injury. This supports the idea that cytokines reflect the acute inflammatory phase of the disease. Their elevation may be more useful for understanding disease activity than for distinguishing kidney-specific injury alone. Meanwhile, levels of TNF-α were also elevated in PSGN (8.11 ± 1.19 pg/mL) compared to controls (3.74 ± 1.4 pg/mL), *p* < 0.005 [124]. In addition, the Th17/IL-17 pathway has been implicated in the maintenance of complement-amplified glomerular injury, particularly through the promotion of neutrophil-predominant inflammation and the maintenance of proliferative lesions. Interestingly, a recent case of infection-associated glomerulonephritis in a child with IL-17RA deficiency demonstrated severe endocapillary glomerulonephritis with marked complement involvement, highlighting the interplay between cytokines and complement [125]. Cohorts of pediatric PIGN patients also provide clues to the profiles of elevated levels of split products of C5b-9 in the acute phase that decrease during the course of recovery in AtCVID (autoimmunity-associated common variable immunodeficiency) patients. Additionally, some patients in AtCVID, especially those who exhibit dysregulated complement levels, are associated with autoantibodies against the components of the activating enzyme in AtCVID patients [122,123]. Moreover, one study in the pediatric population showed a high prevalence of anti-factor B antibodies in children who developed acute post-infectious GN, supporting the role of the alternative pathway in the mechanism of the disease and implying that the immune response to certain microbial/host antigens might transiently trigger the activation of the alternative pathway [122]. This suggests that post-infectious GN may involve transient immune dysregulation beyond infection alone. The antibody response may amplify alternative pathway activation. This could help explain why disease severity varies between patients. Taken together, PIGN appears to be a self-limited disease in many patients, but the degree of complement and cytokine activation may influence how severe the acute episode becomes. The available biomarkers may therefore be more useful for describing activity than for predicting outcome in all cases.

### 4.7. Diabetic Kidney Disease (DKD)

Current evidence linking complement activation to DKD derives from both clinical observations and experimental models. However, it is important to distinguish associations observed in humans from causal mechanisms primarily demonstrated in preclinical studies. Strength of evidence is not the same in humans and in animal models. In patients with diabetic kidney disease, numerous studies have found elevated plasma and urinary concentrations of complement components, including mannose-binding lectin, C4d, Bb, C3a, C5a, and soluble C5b-9. All these studies found significant correlations with proteinuria, histologic severity, and eGFR loss. Similarly, studies on kidney biopsies have found increased levels of C5b-9 in glomerular and tubular compartments and have found associations with interstitial fibrosis [126,127]. While these findings support a link between complement activation and disease severity, they do not establish whether complement activation is a driver of injury or a secondary response to ongoing renal damage. This is an important limitation of the human data. Elevated complement markers may reflect disease activity rather than cause it. Therefore, the biomarkers are informative, but not necessarily causal.

Experimental studies present strong evidence for the potential causal involvement of complement in the pathogenesis of DKD. In streptozotocin-induced or transgenic diabetic models, overexpression of complement components (C3, C4, and C5b-9) precedes structural kidney damage, and genetic or pharmacologic inhibition of these components reduces renal injury [128,129]. This timing supports a possible causal role in experimental DKD. It suggests that complement activation may occur early in disease development. However, this pattern still needs confirmation in human disease. Mechanistically, hyperglycemia appears to activate the alternative pathway by inducing overexpression of complement factor B in podocytes, possibly via mTORC1 signaling [130]. This connects metabolic stress with immune activation. It also suggests that complement is part of the inflammatory response to chronic hyperglycemia. In this sense, DKD is not only a metabolic disorder but also an inflammatory one. In addition, pro-inflammatory cytokines, including IL-6 and IL-17A, have been suggested to play a role in augmenting complement-induced renal inflammation [131]. IL-17A, in particular, contributes to diabetic kidney injury. IL-17−/− mice or animals treated with anti-IL-17 antibodies show significantly reduced albuminuria, glomerular injury, macrophage infiltration, and fibrosis at 12 and 24 weeks (*p* < 0.05–0.001) [132]. However, the applicability of these experimental studies to the pathogenesis of human DKD is uncertain, while direct evidence for causality is still lacking. This is why the translational value of these findings remains limited. Animal studies support a mechanism, but they do not prove that the same process drives human DKD. Human validation is still needed.

Overall, complement activation in DKD should currently be viewed as a marker of disease severity and a possible contributor to progression. The evidence is strongest for association, but weaker for causality in humans. This makes DKD an important but still incompletely defined complement-mediated disease.

## 5. Agents Targeting the Complement System in Kidney Diseases

### 5.1. Eculizumab

The intravenous humanized monoclonal antibody eculizumab binds with high affinity to complement component C5, preventing its proteolytic cleavage into active fragments. Eculizumab inhibits the formation of both anaphylatoxin C5a and membrane attack complex C5b-9 [133]. In patients with complement-mediated thrombotic microangiopathy or atypical hemolytic uremic syndrome, eculizumab-mediated blockade of the terminal pathway has been shown to result in the immediate normalization of hematologic parameters and improvement in renal function over time in studies [134]. Since eculizumab blocks the formation of the membrane attack complex (MAC), it significantly increases susceptibility to invasive meningococcal and other encapsulated organism infections. Pre-treatment meningitis prophylaxis with the meningococcal vaccine is recommended for all patients [135,136]. Long-term C5 blockade requires continued infection vigilance because meningococcal disease can occur despite vaccination [137]. A meta-analysis by Jiang et al. found an increased risk of urinary tract infection (RR 1.33) and severe bacteremia (RR 2.31) with eculizumab. Eculizumab remains the archetypal proof-of-concept for terminal complement blockade. However, infection burden, cost, and incomplete control of upstream activation limit its generalizability across complement-mediated kidney diseases [138]. Critically, this means that eculizumab is best viewed as a treatment for selected high-risk endotypes rather than as a universal option for all complement-mediated kidney diseases.

### 5.2. Ravulizumab

Intravenous ravulizumab was developed from eculizumab through the implementation of the Fc modification approach to decelerate the rate of elimination by the target-mediated mechanism [139]. In a Phase 3 trial of adult patients with atypical hemolytic uremic syndrome (aHUS) (NCT02949128), ravulizumab demonstrated statistically significant efficacy in hematologic and renal parameters. By day 183, treatment-naïve patients showed normalization of platelet count in 83.9%, normalization of lactate dehydrogenase (LDH) in 76.8%, and ≥25% reduction in creatinine in 58.9%. In addition, 68.1% showed an improvement of ≥1 stage in estimated glomerular filtration rate (eGFR). The median time to hematologic response was approximately 30–32 days. The safety profile of the drug was consistent with its mechanism of action, i.e., the blockade of the terminal complement pathway, and no unusual safety issues were noted, with dosing administered at 8-week intervals [140]. The safety profile was consistent with terminal complement inhibition, and no unexpected adverse events were observed. Its extended dosing interval (every 8 weeks) reduces treatment burden. Long-term data indicate sustained efficacy and good tolerability, although standard infection prophylaxis remains necessary [141]. Its longer dosing interval reduces treatment burden, and US cost-minimization modeling suggested 32.4–35.5% lower costs than eculizumab, although real-world nephrology data are still accumulating [142,143]. The main advantage of ravulizumab is its pharmacokinetic properties rather than its mechanism of action. This suggests that its clinical value depends mainly on improving treatment logistics, whereas the underlying biological limitations remain essentially unchanged. Therefore, its clinical impact depends on the same biological assumptions as eculizumab.

### 5.3. Pegcetacoplan

Pegcetacoplan is a subcutaneous pegylated cyclic compstatin analog that binds to native C3 and surface-bound C3b. It prevents the function of C3 and C5 convertases by inhibiting the cleavage of C3 and the generation of C3a/C3b, C5a, and MAC [144]. In a randomized Phase 3 PEGASUS trial of patients with paroxysmal nocturnal hemoglobinuria, pegcetacoplan was found to be superior to eculizumab with respect to changes in hemoglobin concentration, with a least squares mean difference of 3.84 g/dL (*p* < 0.001). Furthermore, 85% of pegcetacoplan-treated patients were transfusion-free compared with 15% of eculizumab-treated patients [90]. In a Phase 3 VALIANT trial (NCT05067127), pegcetacoplan was found to result in a statistically significant reduction in proteinuria compared to placebo in patients with native or recurrent C3G and primary IC-MPGNA (Immune Complex–Mediated Membranoproliferative Glomerulonephritis) 68.1% reduction in urine protein-to-creatinine ratio was observed, and more patients achieved combined renal endpoints without increased rates of serious adverse events or infections [145,146]. However, it did not show any histological improvement and therefore, the long-term implications are unknown. This discordance between proteinuria and histology suggests that short-term surrogate endpoints may overestimate true disease modification. Furthermore, pegcetacoplan was not associated with more adverse events than placebo, and no serious infections from encapsulated bacteria occurred, but early complement blockade still warrants careful infection monitoring in practice. Targeting C3 represents a more proximal intervention but also introduces broader immunological consequences. This creates a trade-off between deeper pathway control and increased risk of infection. As a result, patient selection becomes more critical than with terminal pathway inhibitors.

### 5.4. Iptacopan (LNP023)

Iptacopan is an oral small-molecule compound that selectively inhibits factor B and, consequently, inhibits the assembly of alternative pathway C3 convertase (C3bBb). Furthermore, it inhibits alternative pathway amplification and spares classical pathway activity [147]. In a Phase 3 APPEAR-C3G study (NCT04817618), iptacopan showed significant reductions in proteinuria and favorable normalization/improvements in complement biomarkers compared to placebo. This supports the strategy of an AP-selective, oral convertase-targeting therapy in C3-related glomerulopathies [90]. Similarly, in an APPLAUSE-IgAN trial (NCT04578834), it reduced 24 h UPCR (Urine Protein-to-Creatinine Ratio) by 38.3% in 9 months (*p* < 0.0001), demonstrating strong antiproteinuric effects [148]. As it is an oral inhibitor of factor B, it may be more practical than IV inhibitors. Factor B inhibition highlights the importance of selectively targeting the alternative pathway. This approach may be better aligned with diseases driven by amplification loop dysregulation. Even so, the magnitude of benefit is likely to depend more on the dominant disease endotype than on pathway inhibition alone. However, the long-term safety profile and efficacy of kidney benefits need to be shown for wider use in daily practice [148,149].

### 5.5. Danicopan (ACH-4471)

Danicopan is an oral drug that inhibits the enzymatic activity of factor D, which is the rate-limiting serine protease required to assemble alternative pathway C3 convertase. It remains a phase 2 proof-of-concept therapy for C3G and IC-MPGN, so its efficacy and safety should still be regarded as preliminary. In two Phase 2 studies of danicopan (NCT03369236 and NCT03459443), the first study showed a partial reduction in alternative pathway activity and a >30% reduction in proteinuria in selected patients, but changes in estimated glomerular filtration rates and biopsy scores were not significant [150]. The second trial was terminated early due to insufficient efficacy signals, but no new safety concerns were identified [151]. In addition, these studies provided preliminary evidence of improvements in proteinuria and histological findings [152]. Its clinical development status means that its availability and cost-effectiveness in the field of nephrology cannot yet be established. The variability of response to factor D inhibition suggests that not all patients share the same dominant disease mechanism. This reinforces the need for biomarker-guided stratification. Without such stratification, therapeutic effects may appear inconsistent across studies. This pattern supports a more cautious interpretation of the available evidence, because partial responses do not necessarily imply uniform biological failure.

### 5.6. Avacopan (CCX168)

Avacopan is an oral antagonist of the human C5aR1 receptor that selectively inhibits C5a-induced neutrophil chemotaxis and activation without affecting terminal complement complex formation. In a Phase 3 ADVOCATE study (NCT02994927), avacopan was shown to be non-inferior to the standard prednisone taper regimen for the induction of remission in ANCA-associated vasculitis and superior for sustained remission at 52 weeks. In the overall study population, the mean change in estimated glomerular filtration rate (eGFR) at week 52 was +7.3 mL/min/1.73 m^2^ in the avacopan group compared with +4.1 mL/min/1.73 m^2^ in the prednisone group. Notably, a subgroup analysis of patients with severe renal impairment at baseline (eGFR ≤ 20 mL/min/1.73 m^2^) demonstrated greater improvements in kidney function, with a mean eGFR increase of +16.1 mL/min/1.73 m^2^ in the avacopan group versus +7.7 mL/min/1.73 m^2^ in the prednisone group [153]. The incidence of infection-related serious adverse events was comparable for avacopan and control (13.3% vs. 15.2%) in the ADVOCATE study, thus establishing its acceptable safety profile in the short term. In another randomized phase II study, D. R. W. Jayne et al. evaluated avacopan at a dose of 30 mg twice daily. Treatment response at week 12 was defined as a reduction of at least 50% in BVAS without deterioration. Among patients who received avacopan combined with a reduced prednisone regimen, 86.4% achieved this response. In the avacopan-alone group, 81.0% of patients met the response criteria. By comparison, only 70.0% of patients in the control group receiving high-dose prednisone achieved a similar response. The avacopan regimens met the predefined criteria for non-inferiority of the treatments (*p* = 0.002 and *p* = 0.01, respectively). The 30 mg twice-daily regimen provided virtually complete target coverage [154]. However, the annualized cost of avacopan is estimated to be approximately $75,051, so its availability cannot be taken for granted, even though it can be taken orally [155]. This is the first selective inhibitor of the C5aR1 [156]. Functionally, this makes avacopan a useful example of how blocking inflammatory signaling can be clinically relevant even without full terminal complement inhibition.

### 5.7. Cemdisiran (N-Acetylgalactosamine or GalNAc-Conjugated siRNA)

Cemdisiran is a subcutaneous RNA interference agent that targets the mRNA for hepatic C5 synthesis, resulting in a significant reduction in circulating C5. In a randomized Phase 2 study of patients with IgA nephropathy (NCT03841448), treatment with cemdisiran resulted in an over 95% reduction in circulating C5, leading to a significant reduction in proteinuria. Furthermore, cemdisiran reduces the placebo-adjusted 24 h UPCR by 37.4% at week 32 and serum C5 levels by 98.7%. Thus, targeting the production of C5 from the liver is an important approach for the treatment of renal diseases [157]. However, the study was small, so its long-term efficacy, infection risk, and real-world applicability remain to be established. At this stage, the main importance of cemdisiran is proof of feasibility rather than proof of durable renal benefits.

### 5.8. Crovalimab

Crovalimab is a subcutaneous anti-C5 monoclonal antibody engineered with an Fc region that facilitates low-volume subcutaneous administration. In late-stage PNH studies, including phase 3 randomized studies, crovalimab was non-inferior to an intravenous control for the suppression of hemolysis (COMMODORE-1/COMMODORE-2) [158,159]. In the COMMODORE 1 study (patients previously treated with eculizumab), switching to crovalimab maintained disease control and sustained terminal complement inhibition through 24 weeks, with clinical efficacy comparable to eculizumab and no new safety concerns. In the COMMODORE 2 study, which included complement inhibitor–naïve patients, hemolysis control was achieved in 79.3% of the crovalimab group and 79.0% of the eculizumab group. Transfusion avoidance occurred in 65.7% of patients who received crovalimab and 68.1% of those who received eculizumab. Breakthrough hemolysis was reported in 10.4% of the crovalimab group compared to 14.5% of the eculizumab group. These results demonstrate the noninferiority of crovalimab to eculizumab. Across COMMODORE studies, crovalimab showed safety comparable to eculizumab, but transient immune-complex reactions occurred in 19% of switched patients and were generally mild to moderate. The phase 3 studies COMMUTE-a and COMMUTE-p (NCT04861259/NCT04958265) are targeting patients with atypical hemolytic uremic syndrome (aHUS) [160]. Its low-volume subcutaneous dosing regimen every 4 weeks may also make this agent more convenient and applicable in real life, although long-term data outside PNH remain limited.

Crovalimab demonstrates that alternative delivery strategies can improve treatment accessibility. However, improved convenience does not resolve underlying biological heterogeneity. Thus, clinical benefits remain dependent on appropriate patient selection.

### 5.9. Sutimlimab

Sutimlimab is an intravenous humanized monoclonal antibody that targets C1s, a protease involved in the classical pathway. The efficacy of sutimlimab has been proven in randomized pivotal studies for cold agglutinin disease (NCT03347396), a form of hemolytic anemia resulting from activation of the classical pathway [161]. Studies have shown that blockade of the classical pathway is effective via the proposed pathway, resulting in relief from symptoms associated with CP-induced hemolytic anemia and a positive short-term safety/tolerability profile. Sutimlimab blocks the classical pathway, so vigilance for infections with encapsulated bacteria remains important even though prophylactic vaccination was used in trials [162,163,164]. In terms of cost-effectiveness, the ICER (Incremental Cost-Effectiveness Ratio) is estimated at $2.34 million per QALY (Quality-Adjusted Life Year). Thus, access and cost are major issues for this drug in real-life practice [165]. This illustrates that pathway specificity does not automatically translate into broad clinical utility when the evidence base is narrow, and implementation costs are high.

### 5.10. Narsoplimab

Intravenous narsoplimab targets the lectin pathway by selectively inhibiting the MASP-2 component of the lectin pathway. Phase 2 trials of patients with IgA nephropathy and thrombotic microangiopathy following hematopoietic cell transplantation demonstrated positive results for proteinuria and stable eGFR in some groups [166]. However, in more advanced stages of renal disease, the results have not always been consistent. To precisely define the efficacy of all lectin pathway-related renal diseases, large-scale randomized studies are required. Until such data are available, its role should be considered exploratory rather than practice-changing.

### 5.11. Ruxoprubart

NM8074, or ruxoprubart, is an intravenous humanized monoclonal antibody that selectively targets the catalytic domain of the Bb component of the alternative pathway of the complement system, thereby preventing C3 convertase assembly and inhibiting alternative pathway activity. This agent targets the alternative pathway of the complement system, distinct from C3 or C5 inhibitors, and is of particular importance in the management of kidney disease, including C3 glomerulopathy and aHUS [167]. A Phase Ib, open-label, dose-escalation clinical trial (NCT05647811) is currently underway to evaluate NM8074 in adult patients with C3G. This study assessed the safety, tolerability, immunogenicity, and preliminary efficacy. This trial represents one of the first Bb–specific factor biologics to enter late early-phase clinical evaluation for renal complement–dependent nephropathies [167]. This specificity is attractive, but its true clinical value will depend on whether it improves outcomes beyond what broader upstream blockade can achieve. Other registered studies of NM8074 include atypical hemolytic uremic syndrome (NCT05684159) and ANCA-associated vasculitis (NCT06226662) [168,169]. Because no mature outcome data are yet available, its long-term infection risk, cost, and role in nephrology should be interpreted cautiously.

### 5.12. Sefaxersen

Sefaxersen is a subcutaneous antisense oligonucleotide that has been engineered to decrease hepatic mRNA for complement factor B, leading to a reduction in the activity of the alternative pathway. In human studies, sefaxersen has been shown to significantly reduce systemic factor B levels. This has been correlated with biologically significant effects that have justified continued development of sefaxersen for use in nephrology. In randomized, double-blind, placebo-controlled Phase 1 trials, healthy volunteers received subcutaneous sefaxersen. After a single dose, the mean systemic factor B levels were reduced by up to 38% compared with placebo. Repeated dosing over six weeks resulted in reductions of up to 69%. No clinically meaningful safety signals were observed in laboratory chemistry, hematology, electrocardiography (ECG), or vital signs [170]. The pharmacodynamic effects of sefaxersen have justified its continued development for use in the treatment of high-risk IgA nephropathy, for which an ongoing Phase 3 study, IMAGINATION (NCT05797610), has been initiated. The endpoints for sefaxersen include changes in proteinuria (UPCR) and eGFR. The results of the Phase 2 studies showed that sefaxersen has achieved a mean reduction of 43% in proteinuria at 29 weeks compared to baseline in treated patients [171]. However, this is still a single-arm trial and should be interpreted cautiously until the efficacy and safety of this agent are confirmed in a randomized phase 3 trial. Single-arm reductions in proteinuria are encouraging, but they are not enough to establish a durable class effect or a clear treatment sequence advantage.

**Figure 1:**
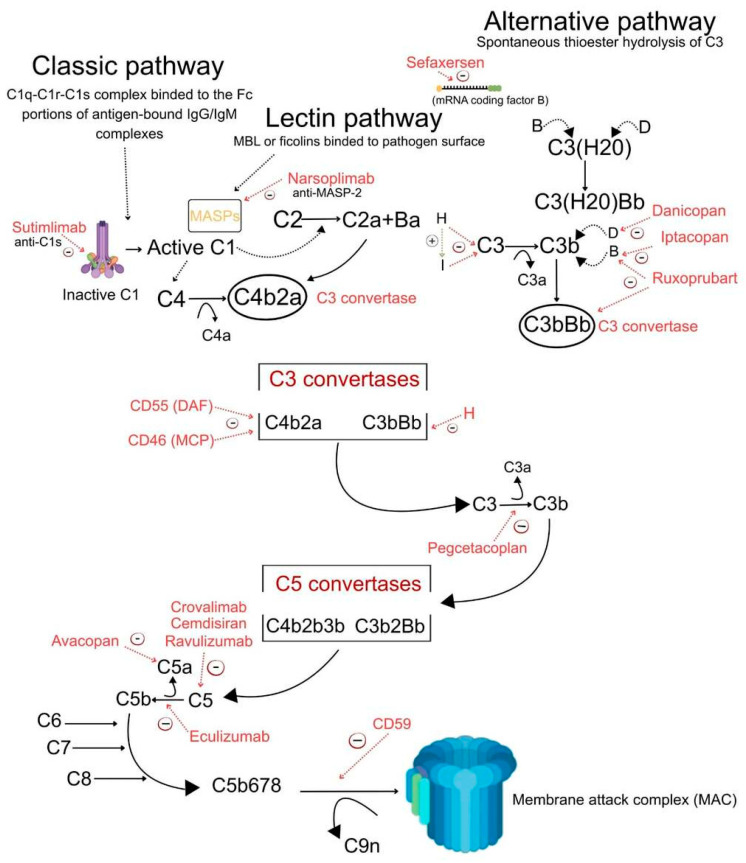
Complement activation pathways and sites of action of complement-targeted therapies.

The aforementioned drugs targeting components of the complement system are summarized in Table 2 and Figure 1.

The complement system is activated through three major pathways: the classical, lectin, and alternative pathways. The classical pathway is initiated by binding of the C1q–C1r–C1s complex to antigen–antibody (IgG or IgM) immune complexes, whereas the lectin pathway is triggered by mannose-binding lectin (MBL) or ficolins recognizing pathogen surfaces and activating MASP-1 and MASP-2. The alternative pathway is constitutively active due to spontaneous hydrolysis of C3 and is amplified via factor B and factor D–dependent formation of the C3bBb C3 convertase. All three pathways converge at the level of C3 convertases (C4b2a and C3bBb), leading to cleavage of C3 into the effector molecules C3a and C3b, generation of C5 convertases, and subsequent cleavage of C5 into C5a and C5b. C5b initiates assembly of the membrane attack complex (MAC; C5b–9), which mediates target cell lysis. The figure illustrates therapeutic agents that regulate distinct steps of complement activation, which include regulators of C1s, MASP-2, factor B, factor D, C3, C5, and C5a. Some of the drugs are depicted as they act on distinct steps of the complement cascade. Abbreviations: C1q—complement component 1q; C1r—complement component 1r; C1s—complement component 1s; MBL—mannose-binding lectin; MASP—MBL-associated serine protease; MASP-2—MBL-associated serine protease 2; C2—complement component 2; C4—complement component 4; C4a—complement component 4a; C3—complement component 3; C3a—complement component 3a (anaphylatoxin); C3b—complement component 3b; Bb—activated fragment of complement factor B; Ba—inactive fragment of complement factor B; D—complement factor D; H—complement factor H; C5—complement component 5; C5a—complement component 5a (anaphylatoxin); C5b—complement component 5b; C6—complement component 6; C7—complement component 7; C8—complement component 8; C9—complement component 9; MAC—membrane attack complex; DAF (CD55)—decay-accelerating factor; MCP (CD46)—membrane cofactor protein; CD59—protectin.

Proximal and terminal complement inhibitors differ substantially in their mechanisms and their clinical significance. Proximal inhibitors, such as inhibitors of C3 or the alternative pathway, have a general influence on the regulation of complement activation, whereas terminal inhibitors (targeting C5 or C5a signaling) specifically regulate the formation of the membrane attack complex. Such differences may have major clinical implications for certain disease entities in which the regulation of the complement system is chronically disordered. Comparison of proximal and terminal complement inhibition is summarized in Table 3.

## 6. Clinical Treatment Algorithm for Complement-Mediated Kidney Diseases

### 6.1. aHUS

In any patient presenting with microangiopathic hemolytic anemia, thrombocytopenia, and renal failure, immediate assessment of ADAMTS13 (A Disintegrin and Metalloproteinase with Thrombospondin Motifs 13) activity is a priority to differentiate aHUS from TTP [172]. In cases with a strong clinical suspicion of complement-mediated thrombotic microangiopathy (TMA) and ADAMTS13 activity >10%, current evidence supports the immediate initiation of terminal complement inhibitors (eculizumab or ravulizumab). This approach may prevent the need for plasmapheresis in severely affected patients and promote rapid hematologic and renal recovery [173,174,175,176]. Investigations of the complement system, including low levels of C3 and normal levels of C4, reduced CH50, and other evidence of alternative pathway activation, can aid in diagnosing complement dysregulation. However, these findings should be interpreted in the context of the patient’s clinical presentation and genetic findings, as these abnormalities can be present in other diseases [177]. Early initiation of complement inhibition in aHUS leads to rapid normalization of platelet counts, reduced hemolysis, and significant improvement in renal function. These effects contribute to sustained remission and may allow for extended dosing intervals of ravulizumab [178]. However, this must be initiated within a specific period only if there are concerns about delaying treatment due to an unacceptable risk to patient health, such as before any procedure where delaying treatment might compromise patient health due to the hypothetical risk of invasive meningitis from C5 inhibition of the C5-NbP (nanobody-based inhibitor) pathway in immunosuppressed patients [179]. The treatment protocol must be adapted and validated based on the kinetics of response to ADAMTS13 and the side effect profile, along with literature-based observations regarding specific side effects, before initiating treatment, genetic mutations, and their relation to C5 inhibitor treatment [180]. Molecular studies of complement regulatory genes (e.g., CFH, CFI, C3, CFB, and MCP) can help establish the presence of complement dysregulation and have prognostic value (e.g., CFH mutations have a worse prognosis and a higher likelihood of relapse). Genetic studies should be considered early in the investigation of aHUS, despite the potential for delayed results [181]. In anti-CFH antibody–associated aHUS, urgent plasma exchange combined with immunosuppression (e.g., corticosteroids, with or without rituximab and/or mycophenolate mofetil) effectively reduces antibody levels, induces remission, and lowers relapse risk. This strategy may be used before or alongside complement inhibition [182]. The main advantage of early C5 blockade in aHUS is the rapid control of thrombotic microangiopathy. Ravulizumab adds the practical benefit of a much longer dosing interval than eculizumab while maintaining similar efficacy and safety [183,184]. The key limitation is that both agents act only at the terminal pathway, so they do not correct the underlying upstream complement dysregulation [184]. Accordingly, C5 inhibition is best positioned as a first-line therapy for complement-mediated aHUS, whereas plasma exchange and immunosuppression remain most relevant in anti-CFH disease or when complement-directed therapy is not immediately available [185].

### 6.2. C3 Glomerulopathy (C3G) and Immune Complex Mediated MPGN (IC-MPGN)

The diagnosis of C3G and IC-mediated MPGN requires the demonstration of prominent C3 deposits in the glomeruli using an electron microscope. In addition, the absence of active infection and monoclonal gammopathy should be established. Simultaneously, the levels of the alternative pathway should be assessed [186]. Optimized supportive care for C3G and IC-mediated MPGN includes the management of proteinuria using renin-angiotensin system inhibitors. Furthermore, blood pressure and lipid levels should be managed. However, the treatment of C3G and IC-mediated MPGN is typically reserved for patients who exhibit signs of progressive proteinuria, inflammatory activity, and decreased eGFR [186]. In cases where convertase and stabilizing autoantibody dysregulation are the primary issues, treatment of the alternative pathway is recommended. Some agents in the alternative pathway include the oral factor B inhibitor iptacopan and the oral factor D inhibitor danicopan [147,152]. In contrast, in cases of severe diffuse C3 deposition in the glomeruli or a pronounced rate of histopathological progression, treatment with a C3 inhibitor targeting the central complement pathway (e.g., pegcetacoplan within clinical trials) may also be considered [187]. Treatment response should be monitored by assessing proteinuria (UPCR), changes in renal function (eGFR), and complement biomarkers (e.g., sC5b-9, Bb, C3d, if available). A repeat kidney biopsy may be considered to evaluate the reduction in glomerular C3 deposition [23,147,188]. The main advantage of proximal complement inhibition in C3G and IC-MPGN is that it more directly targets dysregulation of the alternative pathway compared with terminal C5 blockade. Pegcetacoplan has now demonstrated a greater reduction in proteinuria than placebo. The key limitation is that responses to older regimens and eculizumab have been heterogeneous, and long-term renal outcome data for newer agents are still limited [145,189]. Therefore, complement-targeted treatment is best positioned for progressive, biomarker-supported disease after supportive care has been optimized and secondary causes have been excluded.

### 6.3. IgA Nephropathy

In patients with biopsy-proven IgA nephropathy and sustained proteinuria greater than 1 g/day, or a decrease in eGFR despite maximal medical treatment, complement pathway phenotyping, consisting of the evaluation of the lectin pathway versus the alternative pathway, should be performed [111,190]. If the lectin pathway predominates, inhibition of MASP-2 may be considered. However, the phase 3 ARTEMIS-IgAN trial did not meet its primary endpoints [191]. In patients who exhibit predominantly AP signatures, the use of alternative pathway inhibitors (focusing on oral inhibition of factor B by iptacopan) has led to relevant reductions in proteinuria in randomized trials [192]. Patients with crescents or active histological lesions may require initial immunosuppressive therapy following traditional treatment guidelines, with consideration of complement-directed therapy for refractory disease [193]. Inhibitors of hepatic biosynthesis of C5, such as cemdisiran, have been shown to result in persistent suppression of circulating C5 and have demonstrated promising results for proteinuria outcomes in phase 2 trials of IgAN. Nevertheless, long-term renal outcomes and safety assessments are required [157]. Complement-targeted therapies in IgA nephropathy should currently be considered add-on strategies rather than core treatments for all patients, because their exact position in the therapeutic sequence is still being defined [194,195]. Their use is best guided by clinical, histological, and biomarker features rather than being applied universally. Optimized supportive care remains the foundation of management and includes maximal RAAS (Renin–Angiotensin–Aldosterone System) blockade, blood pressure control, lifestyle modification, and treatment of relevant comorbidities [194]. Complement blockade may also carry safety limitations, particularly an increased risk of serious infections with terminal pathway inhibition [196]. Therefore, complement inhibitors should be positioned later in the algorithm, mainly as phenotype-guided add-on therapy for refractory high-risk patients despite optimized supportive care.

### 6.4. Acute Post-Infectious Glomerulonephritis (APIGN/PIGN)

Most cases of APIGN are self-limiting and associated with transient hypocomplementemia. Evidence highlights transient activation of the alternative pathway and a high prevalence of anti–factor B autoantibodies, particularly in children with active disease [197]. Complement-targeted therapy (e.g., proximal alternative pathway inhibitors or anti-C5 agents) may be considered in atypical cases, especially when C3 levels remain low despite infection clearance. Evaluation of underlying genetic abnormalities and autoantibodies is recommended in such cases [198]. In addition, in unusual chronic or progressive diseases with detectable anti-factor B autoantibodies or abnormalities in convertase regulation, temporary therapy with AP inhibitors may be considered. Alternatively, anti-C5 therapy could also be included as part of comprehensive management algorithms [198]. The main advantage of conventional management of APIGN is that most cases are self-limited and improve with supportive care and infection control [199]. The key limitation of complement blockade is that the evidence remains sparse and is largely restricted to severe or atypical cases. Therefore, AP or C5 inhibition should be considered as rescue therapy for persistent hypocomplementemia, progressive disease, or unusual complement-autoantibody phenotypes rather than as a routine treatment [200,201].

### 6.5. Acute Kidney Injury and Ischemia–Reperfusion

Some studies support the role of locally produced C3 in the initial tubular injury cascade and the role of the C5a-C5aR1 axis in ischemia–reperfusion injury. Thus, the basis for complement inhibition in the prevention of delayed graft function (DGF) is robust, and routine use in this context is supported. However, routine clinical use remains limited due to the lack of randomized trials addressing key outcomes, such as dialysis requirement, time to graft function, and long-term eGFR decline. Therefore, complement inhibition in this setting should be considered experimental [202]. Future trials should include these endpoints, along with pharmacodynamic measures such as urinary levels of sC5b-9 and tissue complement gene expression in the kidney.

### 6.6. Diabetic Kidney Disease (DKD)

Current studies support the notion that classical, lectin, and alternative pathway components/C5b-9 deposits are related to the progression of interstitial fibrosis and the extent of proteinuria in diabetic kidney disease [203]. Another preclinical study on the genetic and loss-of-function aspects of the disease implicated factor B and the enzymatic cascade in the progression of tubulopathy in diabetic animal models [130]. Proteomic analyses of complement activation signatures show promise as predictive biomarkers of disease progression and treatment responses. These markers may enhance the design of clinical trials and serve as pharmacodynamic indicators of complement inhibitor efficacy [204]. Currently, the use of complement inhibitors in DKD remains investigational. Their application should be guided by biomarker enrichment strategies, as well as changes in eGFR and albuminuria. Pharmacodynamic evidence of target engagement should also be demonstrated before broader clinical implementation is considered [203,205,206,207].

The individual therapies for kidney diseases, their advantages, limitations and positioning are summarized in Table 4.

## 7. Future Directions

Future research should precisely define the optimal duration of the complement pathway blockade, as current strategies are largely empirical, and prospective data are limited. Available data suggest that it is not necessary for all patients to have lifelong treatment, as observational data have demonstrated that the relapse rate in 23% to 29.6% of the population is associated with discontinuation of treatment [208]. However, it is essential to emphasize that the relapse rate is not uniform and is influenced by genetic background. The relapse rate in the population harboring the complement pathway mutations is up to 50%, whereas in the population without mutations, the relapse rate is less than 10% [209]. The duration of treatment discontinuation is an important factor in relapse. Studies have shown that stopping treatment for a shorter period (less than 6–12 months) is associated with a higher relapse rate than discontinuation after a longer period (more than 12 months) [210]. Biomarker-guided personalization is likely to be one of the most promising approaches in the future. In aHUS, a higher level of sC5b-9 in plasma at discontinuation of eculizumab therapy was associated with a higher risk of relapse. Rare variants in CFH, MCP/CD46, and C3 also predict recurrence [181,208]. Pathway-specific drugs have already shown that biomarker-enriched therapy can produce measurable effects [140,145,148]. Long-term safety monitoring should remain a requirement, particularly for invasive meningococcal infection, as a real-world pharmacovigilance approach found meningococcal infection rates of 0.25 per 100 patient-years for eculizumab and 0.10 per 100 patient-years for ravulizumab [136]. Long-term monitoring should also include regular follow-up visits, such as every 1 to 3 months in the first year, as research has shown that most relapses tend to occur within the first 12 months following discontinuation of therapy. This will enable timely intervention to resume therapy [210]. Future trials should include biomarkers such as C3, sC5b-9, and complement genes. This approach will enable a more individualized method for determining stopping rules, retreatment strategies, and long-term safety monitoring.

## 8. Conclusions

Dysfunction of the complement system is presented in this review as the main etiological factor and pathogenic mechanism in the spectrum of kidney diseases, including acute kidney injury, immune complex-mediated glomerulonephritis, C3 glomerulopathy, atypical hemolytic uremic syndrome, IgA nephropathy, and diabetic nephropathy. The amplification loop of the alternative pathway and its effector molecule C5 are highlighted in this review as active participants in the pathogenesis of kidney diseases through their pro-inflammatory and pro-renal dysfunction activities. However, some complement molecules, such as Ba, Bb, and soluble C5b-9, have been shown to be correlated with the activity and outcome of kidney pathology and have diagnostic, predictive, and pharmacodynamic monitoring potential. Recent advances in complement-targeted therapeutics, such as C5 inhibitors, C3 inhibitors, and alternative pathway inhibitors, show signs of translation from the current understanding of complement activation and regulation in the management of kidney diseases. The future prospects of complement therapeutics in the management of kidney diseases are expected to be driven by complement phenotyping and the application of pathway-specific therapeutics in combination with biomarker analysis.

## Figures and Tables

**Table 1:** Practical complement biomarker panel for kidney disease monitoring.

Marker	Sample	Suggested Timing	Short Interpretation	Clinical Status and Practical Limitations
sC5b-9 (soluble terminal complex)	Plasma (or urine if validated)	Baseline, post-treatment days 7–14, then monthly while on therapy	Indicates activation of the terminal pathway; used to confirm engagement of targets for C5-directed therapies, as well as to track complement activation.	Closest to clinical implementation, but still limited by inter-assay variability and lack of full standardization. Availability is restricted to specialized laboratories; moderate-to-high cost.
Ba (factor B fragment)	Urine (or plasma)	Peri-injury/AKI baseline and early post-op (hours–days)	Serves as an early biomarker of alternative pathway activation in acute kidney injury, potentially preceding increases in creatinine, which can help to triage high-risk patients.	Primarily research-use biomarker. Limited assay standardization, variable availability, and insufficient validation for routine clinical use.
Bb	Plasma	Baseline and interval (monthly)	Represents activity of the alternative pathway (AP) convertase, with potential use in selecting alternative pathway-targeting therapies, as well as in pharmacodynamic (PD) assessments.	Research/translational biomarker. Limited availability, lack of standardized thresholds, and inter-laboratory variability constrain clinical use.
C3d/C3c	Plasma or tissue (biopsy)	Baseline and on-therapy biopsy	Represents activity of the alternative pathway convertase, with potential use in selecting alternative pathway-targeting therapies, as well as in pharmacodynamic assessments.	Mainly research-based, especially in tissue. Requires specialized techniques; interpretation is not standardized and limits routine applicability.
Anti-factor H/nephritic factor (C3NeF)	Plasma	Baseline	Indicates acquired drivers (autoantibodies) with potential as biomarkers predicting responses to convertase-directed strategies or the need for immunomodulatory approaches.	Partially available in clinical practice, but with limited standardization. Assays are heterogeneous, availability is center-dependent, and costs may be high.
Complement regulators genetic panel (*CFH*, *CFI*, *C3*, *CFB*, etc.)	Blood (DNA)	Baseline	Indicates high-risk genetic variants, which have potential as biomarkers predicting prognosis, as well as the duration or choice of complement blockade.	Clinically relevant but specialized. Requires genetic expertise for interpretation; relatively high cost and variable accessibility. Variant significance may be uncertain.
Urine complement proteomics (multiplex)	Urine	Baseline (selected intervals in trials)	A combination of biomarkers with potential as an exploratory pharmacodynamic biomarker set, which enriches clinical trials and has correlations with histologic changes.	Exploratory/research-use only. High cost, lack of assay standardization, and limited availability preclude routine clinical implementation.

Abbreviations: AP—alternative pathway; sC5b-9—soluble terminal complex; Ba—factor B fragment; Bb—catalytic subunit of factor B; C3d—complement component 3 degradation fragment; C3c—complement component 3 cleavage fragment; C3NeF—C3 nephritic factor; CFH—complement factor H; CFI—complement factor I; CFB—complement factor B; PD—pharmacodynamics.

**Table 2:** Key complement-targeted agents and representative trials.

Drug	Phase	Acronym	Mechanism	Indication	Trial ID	References
Eculizumab	pivotal studies/registries	–	anti-C5 monoclonal antibody (terminal pathway blockade)	complement-mediated TMA, aHUS	pivotal studies and registry/prospective programs	[133,134,135,136,137,138]
Ravulizumab	Phase 3	–	long-acting anti-C5 monoclonal antibody	aHUS (adults)	NCT02949128	[139,140,141,142,143]
Pegcetacoplan	Phase 3	VALIANT	C3/C3b inhibitor	kidney diseases (C3-driven), PNH	NCT05067127	[144,145,146,147,148]
Iptacopan (LNP023)	Phase 3	APPEAR-C3G; APPLAUSE-IgAN	factor B inhibitor (alternative pathway C3 convertase inhibition)	C3 glomerulopathy; IgA nephropathy	NCT04817618; NCT04578834	[147,148,149]
Danicopan (ACH-4471)	Phase 2	–	factor D inhibitor	C3G; IC-MPGN	NCT03369236; NCT03459443	[150,151,152]
Avacopan (CCX168)	Phase 3	ADVOCATE	C5aR1 antagonist	ANCA-associated vasculitis	NCT02994927	[153,154,155,156]
Cemdisiran	Phase 2	–	GalNAc-conjugated siRNA targeting hepatic C5 synthesis	IgA nephropathy	NCT03841448	[158]
Crovalimab	Phase 3	COMMUTE-a; COMMUTE-p	anti-C5 antibody with engineered Fc	aHUS	NCT04861259; NCT04958265	[158,159,160]
Sutimlimab	Phase 3 (pivotal)	–	anti-C1s monoclonal antibody (classical pathway inhibition)	cold agglutinin disease	NCT03347396	[161,162,163,164,165]
Narsoplimab	Phase 2	–	anti-MASP-2 antibody (lectin pathway inhibition)	IgA nephropathy; post-HCT thrombotic microangiopathy	-	[166]
Ruxoprubart (NM8074)	Phase Ib (and early clinical)	–	anti-factor Bb monoclonal antibody (alternative pathway C3 convertase inhibition)	C3 glomerulopathy; aHUS; ANCA-associated vasculitis	NCT05647811; NCT05684159; NCT06226662	[167,168,169]
Sefaxersen	Phase 1 and Phase 3	IMAGINATION	antisense oligonucleotide targeting hepatic factor B mRNA	high-risk IgA nephropathy	NCT05797610	[170,171]

Abbreviations: TMA—thrombotic microangiopathy; aHUS—atypical hemolytic uremic syndrome; PNH—paroxysmal nocturnal hemoglobinuria; C3G—C3 glomerulopathy; IC-MPGN—immune complex–mediated membranoproliferative glomerulonephritis; ANCA—anti-neutrophil cytoplasmic antibody; HCT—hematopoietic cell transplantation; siRNA—small interfering RNA; GalNAc—N-Acetylgalactosamine; MASP-2—mannose-binding lectin-associated serine protease-2; Bb—catalytic subunit of factor B.

**Table 3:** Comparative Framework of Proximal vs. Terminal Complement Inhibition.

Feature	Proximal Inhibition	Terminal Inhibition
Target level	Upstream components (C3, factor B, factor D)	Downstream component (C5 or C5a/C5aR1)
Examples of drugs	Pegcetacoplan, Iptacopan, Danicopan, Sefaxersen	Eculizumab, Ravulizumab, Avacopan, Crovalimab
Mechanism of action	Blocks the formation of C3 convertase or C3 cleavage → inhibits entire cascade amplification	Blocks cleavage of C5 or C5a signaling → prevents MAC formation and terminal inflammation
Pathway coverage	Broad (affects classical, lectin, and alternative pathways via C3 or AP-specific inhibition)	Narrow (acts only at the terminal pathway stage
Effect on C3 activation	Strong inhibition	No effect
Effect on C5 activation/MAC	Prevented indirectly	Directly inhibited
Impact on upstream inflammation	Reduces early inflammatory mediators	Limited (upstream activation persists)
Disease rationale	Best for diseases driven by alternative pathway dysregulation (e.g., C3G, IgAN)	Effective in diseases with dominant terminal pathway activation (e.g., aHUS, TMA)
Efficacy considerations	May better control disease at the source (upstream dysregulation)	May be insufficient when upstream activation remains active
Infection risk	Potentially broader (due to upstream blockade of opsonization)	High risk of meningococcal infections (due to MAC inhibition)
Advantages	-Targets the root cause of complement activation - Broader pathway control	- Well-established clinical use - Rapid control of severe disease
Limitations	- Less long-term clinical data - Possible higher infection susceptibility	- Does not control upstream dysregulation - Variable efficacy in diseases like C3G
Personalized medicine role	Preferred when biomarkers indicate alternative pathway activation	Preferred when biomarkers indicate dominant C5/MAC activation

Abbreviations: C3—complement component 3; C5—complement component 5; C5a—complement component 5a (anaphylatoxin); C5aR1—complement component 5a receptor 1; MAC—membrane attack complex; AP—alternative pathway; C3G—C3 glomerulopathy; IgAN—IgA nephropathy; aHUS—atypical hemolytic uremic syndrome; TMA—thrombotic microangiopathy.

**Table 4:** Comparative Framework of Therapies for Kidney Diseases.

Disease	Strategy	Advantages	Limitations	Clinical Positioning
aHUS	C5 inhibition (eculizumab, ravulizumab)	Rapid control of TMA; strong evidence base; ravulizumab reduces treatment burden	Does not block upstream complement activation; infection risk; high cost	First-line therapy in suspected complement-mediated aHUS
	Plasma exchange + immunosuppression	Effective in anti-CFH antibody disease; reduces autoantibody levels	Non-specific; slower onset; invasive (PLEX)	Adjunct or alternative in anti-CFH aHUS or when diagnosis is uncertain
	Proximal complement inhibition	Targets upstream dysregulation; potentially more complete pathway control	Limited clinical data in aHUS; unclear long-term outcomes	Emerging option, not yet standard of care
C3G/IC-MPGN	Supportive therapy (RAAS blockade)	Widely available; slows CKD progression; low risk	Does not target the disease mechanism	First-line baseline therapy for all patients
	C5 inhibition	Some benefit in selected cases	Inconsistent efficacy; does not address upstream AP dysregulation	Limited role, selected or refractory cases
	Proximal/AP inhibition (C3, factor B/D)	Mechanistically aligned with AP dysregulation; promising trial results	Limited long-term outcome data; access issues	Preferred targeted therapy in progressive disease
IgA nephropathy	Supportive therapy	Proven benefit on progression; standard of care	Insufficient in high-risk disease	Foundation of treatment
	Immunosuppression	Effective in active inflammatory lesions	Side effects; variable efficacy	Selected patients with active disease
	AP inhibition (e.g., factor B inhibitors)	Reduces proteinuria; oral options available	Long-term renal outcomes unclear	Add-on in high-risk or refractory disease
	Terminal pathway inhibition	Potential benefit in selected phenotypes	Limited evidence; unclear target population	Not routine, investigational or niche use
APIGN/PIGN	Supportive therapy	Most cases are self-limited, effective and safe	None in the typical disease	Standard of care
	Complement inhibition	Strong biological rationale; targets early injury cascade	Lack of robust RCT data; unclear clinical benefit	Experimental/trial-based use
Diabetic kidney disease	Standard therapy (RAAS, SGLT2i, etc.)	Strong evidence; reduces progression risk	Does not directly target the complement	Core therapy
	Complement inhibition	Mechanistic rationale; biomarker-driven potential	Limited human data; unclear efficacy	Investigational, biomarker-guided trials

Abbreviations: aHUS—atypical hemolytic uremic syndrome; C5—complement component 5; TMA—thrombotic microangiopathy; CFH—complement factor H; PLEX—plasma exchange; C3G—C3 glomerulopathy; IC-MPGN—immune complex-mediated membranoproliferative glomerulonephritis; RAAS—renin–angiotensin–aldosterone system; CKD—chronic kidney disease; AP—alternative pathway; IgA—immunoglobulin A; APIGN—acute post-infectious glomerulonephritis; PIGN—post-infectious glomerulonephritis; RCT—randomized controlled trial; SGLT2i—sodium–glucose cotransporter 2 inhibitors.

## Data Availability

No new data were created or analyzed in this study. Data sharing is not applicable to this article.

## Abbreviations

The following abbreviations are used in this manuscript:

AKI	Acute Kidney Injury
aHUS	Atypical Hemolytic Uremic Syndrome
LN	Lupus Nephritis
SLE	Systemic Lupus Erythematosus
C3G	Complement 3 Glomerulopathy
IgAN	IgA Nephropathy
DKD	Diabetic Kidney Disease
PIGN	Post-Infectious Glomerulonephritis
APIGN	Acute Post-Infectious Glomerulonephritis
TMA	Thrombotic Microangiopathy
PNH	Paroxysmal Nocturnal Hemoglobinuria
DGF	Delayed Graft Function
CP	Classical Pathway
LP	Lectin Pathway
AP	Alternative Pathway
MAC	Membrane Attack Complex
sC5b-9	Soluble C5b-9
FH	Factor H
FI	Factor I
DAF	Decay-Accelerating Factor
MCP	Membrane Cofactor Protein
C1-INH	C1 Inhibitor
MBL	Mannose-Binding Lectin
MASP	MBL-Associated Serine Protease
CRD	Carbohydrate Recognition Domain
CCP	Complement Control Protein
Ba/Bb	Factor B fragments
CFH	Complement Factor H
CFI	Complement Factor I
CFB	Complement Factor B
CFHR	Complement Factor H–Related protein
C5aR1	C5a Receptor 1
PI3K	Phosphoinositide 3-Kinase
PLCβ	Phospholipase C beta
IL-6	Interleukin 6
IL-6R	Interleukin 6 Receptor
sIL-6R	Soluble Interleukin 6 Receptor
IL-17A	Interleukin 17A
IL-18	Interleukin 18
IL-1β	Interleukin 1 beta
TNF-α	Tumor Necrosis Factor alpha
TNF-R1	Tumor Necrosis Factor Receptor 1
STAT3	Signal Transducer and Activator of Transcription 3
NF-κB	Nuclear Factor kappa B
Akt	Protein Kinase B
IP_3_	Inositol 1,4,5-trisphosphate
Gi	Inhibitory G protein
Gβγ	G protein beta-gamma subunits
Th17	T helper 17 cells
Treg	Regulatory T cells
EC4d	Erythrocyte-bound C4d
BC4d	B cell-bound C4d
CB-CAPs	Cell-bound Complement Activation Products
DDD	Dense Deposit Disease
C3GN	C3 Glomerulonephritis
IC-MPGN	Immune Complex–Mediated Membranoproliferative Glomerulonephritis
ANCA	Anti-Neutrophil Cytoplasmic Antibodies
rs17611	Reference SNP ID number 17611
TTP	Thrombotic Thrombocytopenic Purpura
ADAMTS13	A Disintegrin and Metalloproteinase with Thrombospondin Motifs 13
AtCVID	autoimmunity-associated common variable immunodeficiency
eGFR	Estimated glomerular filtration rate
UPCR	Urine Protein-to-Creatinine Ratio
ECG	Electrocardiogram
GalNAc	N-Acetylgalactosamine
siRNA	small interfering RNA
ICER	Incremental Cost-Effectiveness Ratio
QALY	Quality-Adjusted Life Year
